# Isothermal Titration Calorimetric Studies on the Interaction of the Major Bovine Seminal Plasma Protein, PDC-109 with Phospholipid Membranes

**DOI:** 10.1371/journal.pone.0025993

**Published:** 2011-10-14

**Authors:** V. Anbazhagan, Rajeshwer S. Sankhala, Bhanu Pratap Singh, Musti J. Swamy

**Affiliations:** School of Chemistry, University of Hyderabad, Hyderabad, Andhra Pradesh, India; University of Oulu, Finland

## Abstract

The interaction of the major bovine seminal plasma protein, PDC-109 with lipid membranes was investigated by isothermal titration calorimetry. Binding of the protein to model membranes made up of diacyl phospholipids was found to be endothermic, with positive values of binding enthalpy and entropy, and could be analyzed in terms of a single type of binding sites on the protein. Enthalpies and entropies for binding to diacylphosphatidylcholine membranes increased with increase in temperature, although a clear-cut linear dependence was not observed. The entropically driven binding process indicates that hydrophobic interactions play a major role in the overall binding process. Binding of PDC-109 with dimyristoylphosphatidylcholine membranes containing 25 mol% cholesterol showed an initial increase in the association constant as well as enthalpy and entropy of binding with increase in temperature, whereas the values decreased with further increase in temperature. The affinity of PDC-109 for phosphatidylcholine increased at higher pH, which is physiologically relevant in view of the basic nature of the seminal plasma. Binding of PDC-109 to Lyso-PC could be best analysed in terms of two types of binding interactions, a high affinity interaction with Lyso-PC micelles and a low-affinity interaction with the monomeric lipid. Enthalpy-entropy compensation was observed for the interaction of PDC-109 with phospholipid membranes, suggesting that water structure plays an important role in the binding process.

## Introduction

The seminal plasma in mammals serves as a carrier of freshly ejaculated spermatozoa through the female genital tract to their final destination, the uterus. During this passage spermatozoa undergo a series of biochemical and ultrastructural changes – collectively referred to as capacitation – a necessary event before they attain the ability to fertilize the egg [1], [2]. It has been established that certain seminal plasma proteins inhibit inappropriate acrosomal reaction whereas other proteins bind to the surface of spermatozoa and induce their capacitation [3], [4]. Among the various mammalian species proteins of bovine seminal plasma have been studied in great detail. The major protein fraction of bovine seminal plasma is composed of four acidic proteins designated as BSP-A1, BSP-A2, BSP-A3 and BSP-30 kDa, which are collectively referred to as bovine seminal plasma proteins, or as BSP proteins [5], [6]. BSP-A1 and BSP-A2 are identical in their primary structure and differ only in the degree of glycosylation and their mixture is also referred to as PDC-109. Homologues of BSP protein are also present in the seminal plasma of other mammalian species such as stallion, pig, goat etc [7]–[11]. These observations show that the BSP family of proteins are widely distributed in mammalian seminal plasma, exist in several forms in each species and may play a common biological role. Recent studies show that spermatozoa of several mammals such as bull, pig, rabbit etc. bind with the surface of mucosal epithelium by sperm surface proteins, leading to the formation of oviductal sperm reservoir, which maintains the sperm fertile and hyper motile for longer duration, which helps in successful fertilization [12], [13].

PDC-109 is the major protein of bovine seminal plasma and is present at 15–25 mg/mL concentration in it [14]. It is a polypeptide with 109 amino acids and contains a 23-residue *N*-terminal stretch, followed by two tandemly repeating fibronectin type-II (Fn-II) domains [15]–[17]. Upon ejaculation, around 9.5 million PDC-109 molecules bind to each sperm cell [18]. This interaction is mediated by the binding of PDC-109 to choline phospholipids such as phosphatidylcholine (PC) and sphingomyelin, present on the outer leaflet of the sperm plasma membrane [19]. Each Fn-II domain binds to one choline phospholipid molecule on the sperm plasma membrane and stimulates extracellular efflux of cholesterol and phospholipids (termed as *cholesterol efflux*), which is an important step in sperm capacitation [20], [21]. Single crystal X-ray diffraction studies have shown that both the choline phospholipid binding sites of PDC-109 are on the same face of the protein molecule [22]. Biophysical studies have shown that although PDC-109 exhibits high specificity for choline phospholipids, it also recognizes other phospholipids such as phosphatidylglycerol and phosphatidylserine, albeit with considerably lower affinity [23]–[26]. Spin-label ESR studies indicate that, upon binding to phosphatidylcholine membranes, PDC-109 penetrates into the hydrophobic interior of the membrane up to the 14^th^ C-atom of the lipid acyl chains and that cholesterol increases the selectivity of the protein for different phospholipids [23], [24]. The higher affinity of PDC-109 for choline phospholipids could be explained in terms of faster association and slower dissociation rate constants for phosphatidylcholine as compared to other phospholipids [25]. Fluorescence spectroscopic studies are consistent with these observations and suggest that upon binding to lipid membranes a segment of the polypeptide chain containing Trp-90 penetrates deep into the membrane interior [27].

PDC-109 appears to be a multi-functional protein as it binds – besides choline phospholipids – to a variety of other, structurally unrelated molecules. Interaction of this protein with Le^a^ trisaccharide, present on the surface of the oviductal epithelium in the cow, has been suggested to help in the formation of sperm reservoir in the oviduct [28], [29]. Binding of PDC-109 and the other BSP proteins to heparin forms the basis of an affinity chromatographic method for their purification [6], [30]. In addition, PDC-109 has been reported to recognize a number of other biomolecules such as D-fructose, different types of collagen, fibrinogen and apolipoprotein A-1 [31]–[33]. Very recent work shows that PDC-109 exhibits chaperone-like activity against a variety of target proteins *in vitro*, which could be modulated by phospholipid binding [34], [35]. These observations suggested that PDC-109 may function as a molecular chaperone *in vivo* and help in maintaining other seminal plasma proteins in a functionally active, folded form.

The interaction of PDC-109 with membranes made up of different phospholipids has been investigated by a variety of biophysical techniques and some aspects of the thermodynamic forces governing its interaction with phosphatidylcholine membranes have been reported [23]–[27], [36]–[38]. However, a detailed understanding of the role of hydrophobic interaction in the binding and how it is modulated by temperature and pH of the surrounding environment has not been reported so far, although it was recently reported that the binding of this protein with palmitoleoylphosphatidylcholine (POPC) membranes is endothermic at low temperature and exothermic at high temperature [38]. In the present study, we have utilized isothermal titration calorimetry (ITC) to derive detailed information on the energetics of PDC-109 binding to lipid bilayers. The binding of PDC-109 to membranes containing phosphatidylcholine was observed to be stronger than to phosphatidylglycerol membranes, which is consistent with the results obtained in our earlier SPR studies [25]. The effect of the presence of cholesterol on the interaction of this protein with phosphatidylcholine membranes was also investigated as a function of temperature. ITC studies were also performed on the interaction of PDC-109 with a single chain phospholipid, lysophosphatidylcholine (Lyso-PC) and the data obtained were analyzed in terms of two types of binding interactions between the protein and the ligand. PDC-109 exhibited a stronger affinity for phosphatidylcholine at higher pH, which could be of physiological significance in view of the basic nature of seminal plasma.

## Materials and Methods

### Materials

Phosphorylcholine chloride (Ca^2+^ salt), choline chloride and tris(hydroxymethyl)amino–methane (*Tris base*) *were* obtained from Sigma (St. Louis, MO, USA). Sephadex G-50 (superfine) and DEAE Sephadex A-25 were purchased from Pharmacia Biotech (Uppsala, Sweden). Dimyristoylphosphatidylcholine (DMPC), dipalmitoylphosphatidylcholine (DPPC), dimyristoylphosphatidylglycerol (DMPG), dimyristoylphosphatidylethanolamine (DMPE), Lyso-PC and cholesterol were obtained from Avanti Polar Lipids (Alabster, AL, USA). All other chemicals used were of the highest purity available from local suppliers.

#### Purification of PDC-109

PDC-109 was purified from the seminal plasma of healthy and reproductively active Ongole bulls by gel filtration on Sephadex G-50, followed by affinity chromatography on DEAE Sephadex A-25 as described earlier [23], [39] and the protein obtained was found to be pure by SDS-PAGE [40] (see Fig. S1). Concentration of purified PDC-109 was estimated from its A_280 nm_ value of 2.5 for 1 mg/ml protein [39].

#### Turbidimetric studies

Binding of PDC-109 to phospholipid multilamellar vesicles was investigated by turbidimetry. Sample turbidities were monitored by measuring the optical density at 333 nm in an Analytik Jena Spekol-1200 UV-Vis single beam spectrometer in a 1 cm pathlength cell. Samples were prepared by taking a small, weighed quantity of the lipid (DMPC, DMPG or DMPE) in a glass test tube, dissolving it in ca. 50–100 µL of dichloromethane (or dichloromethane/methanol mixture), followed by removing the solvent under a stream of nitrogen gas. The resulting thin lipid film was vacuum desiccated for a minimum of 4 hours to completely remove the solvent and then hydrated with an appropriate volume of 50 mM Tris-HCl buffer containing 150 mM NaCl, 5 mM EDTA and 0.025% NaN_3_, pH 7.4 (TBS-I) to yield the final desired concentration. The samples were then vortexed and subjected to 8 freeze-thaw cycles to give a suspension of multilamellar vesicles (MLVs). Sample turbidity was then measured for the lipid dispersion as well as upon addition of different amounts of PDC-109 from a high concentration stock solution.

### ITC studies on the binding of PDC-109 to diacyl phospholipids

The interaction of PDC-109 with different phospholipids such as DMPC, DMPG or DPPC was investigated by isothermal titration calorimetry using a MicroCal VP-ITC instrument (MicroCal LLC, Northampton, MA, USA). A thin uniform layer of the appropriate phospholipid was prepared in a glass test tube as described above and hydrated with the appropriate buffer to yield the desired final concentration of the phospholipid. This suspension was subjected to bath sonication until a clear solution was obtained, indicating the formation of unilamellar vesicles. Solutions were degassed under vacuum prior to use in ITC experiments. Binding of PDC-109 to phospholipid vesicles was studied at various temperatures as indicated in Table 1. Typically, 25 consecutive injections of 5 µL aliquots of the protein at a concentration 250 µM were added with the help of a rotator stirrer-syringe into the calorimeter cell of 1.445 mL filled with 80 µM of the lipid in vesicular form. To minimize the contribution of heat of dilution to the measured heat change, the protein solution and lipid vesicles were prepared in the same buffer. Injections were made at intervals of 3 minutes for all titrations except for those involving DMPG, for which the interval between successive injections was 15–20 minutes. In order to ensure proper mixing after each injection, a constant stirring speed of 300 rpm was maintained during the experiment. Control experiments were performed by injecting PDC-109 solution into the buffer solution in an identical manner and the resulting heat changes were subtracted from the measured heats of binding. Since the first injection is often inaccurate, a 1 or 2 µL injection was added first and the resultant point was deleted before the remaining data were analyzed using a ‘*one set of sites*’ binding model as described below.

**Table 1:** Thermodynamic parameters obtained by isothermal titration calorimetry for PDC-109 binding to phsophotidylcholine and phosphatidylglycerol unilamellar vesicles.

Temperature (°C)	*n*	*K*_a_×10^−5^ (M^−1^)	Δ*G* (kcal.mol^−1^)	Δ*H* (kcal.mol^−1^)	Δ*S* (cal.mol^−1^.K^−1^)
DPPC					
20	18.2 (±3.3)	0.8 (±0.3)	−6.57	0.5 (±0.1)	23.7 (±0.8)
25	14.4 (±1.0)	1.3 (±0.4)	−6.98	0.9 (±0.4)	26.5 (±1.0)
30	7.6 (±1.2)	2.2 (±0.4)	−7.41	5.1 (±0.9)	41.1 (±2.5)
36	7.2 (±0.1)	20.7 (±8.9)	−8.93	5.1 (±0.5)	45.2 (±2.5)
39*	6.5 (±0.1)	24.5 (±6.5)	−9.12	5.5 (±0.1)	46.7
DMPC					
10	21.6 (±0.2)	3.9 (±0.8)	−7.24	1.2±0.1	29.7 (±0.4)
15	13.4 (±0.9)	3.5 (±0.6)	−7.31	2.3±0.2	33.3 (±0.5)
20	7.2 (±0.3)	4.1 (±1.5)	−7.52	4.5±0.5	40.8 (±1.8)
DMPG					
7.4	55	0.26 (±0.03)	−5.91	2.9 (±0.6)	30.0 (±2.4)

*Data from a single measurement.

Values reported are averages of results obtained from 2–4 independent experiments with standard deviations indicated in parentheses.

#### Binding of PDC-109 to Lyso-PC micelles

The interaction between PDC-109 and Lyso-PC was also investigated by ITC at 25°C. In these experiments, 5 µL aliquots of a 10.88 mM solution of Lyso-PC were added to the calorimeter cell containing 0.4–0.45 mM of protein at an interval of 200 seconds. Control titrations were performed by injecting Lyso-PC into the buffer and the resulting blank titration data were subtracted from the titration data obtained with PDC-109. The corrected data thus obtained were analyzed using a ‘*two sets of sites*’ binding model.

### Effect of cholesterol on the interaction of PDC-109 with DMPC vesicles

The effect of cholesterol on the interaction between phosphatidylcholine and PDC-109 was investigated by titration calorimetric experiments at various temperatures. Lipid samples for these experiments were prepared as follows. Appropriate amounts of DMPC and cholesterol were weighed accurately into a glass test tube to give 25 mol% of the sterol in the mixture and dissolved in a minimum volume of dichloromethane/methanol mixture and samples were prepared by following the procedure described above. ITC studies were carried out at different temperatures by placing the lipid/cholesterol mixture in the sample cell and protein in the syringe. Each titration consisted of 20 injections (5 µL each) of the protein (500 µM) in the syringe into the cell of 1.445 mL filled with 76 µM of the phospholipid. Control titrations were performed in a similar manner as described above.

#### Effect of pH on the interaction of PDC-109 with phospholipid membranes

PDC-109 was dialyzed against 50 mM Tris buffer containing 150 mM NaCl and 5 mM EDTA, at different pH (7.4–9.0) and diluted with the same buffer to a final working concentration of 220–250 µM. To minimize the contribution to binding heat from dilution, the protein solution and the unilamellar vesicles were prepared in the same buffer. ITC experiments were performed essentially as described above by injecting the protein from the syringe into the calorimeter cell containing ca. 90–100 µM of DMPC or DMPG. Control titrations were performed by injecting the protein into the buffer of appropriate pH. The data were analysed by fitting the experimental values to the ‘*one set of sites*’ binding model described above.

#### Analysis of ITC data

The data obtained from the above calorimetric titrations were analyzed using the Origin ITC data analysis software supplied by the instrument manufacturer [41]. Data obtained for the interaction of PDC-109 with diacylphospholipids could be fit satisfactorily using the ‘*one set of sites*’ binding model, whereas the binding data obtained for the interaction of PDC-109 with Lyso-PC could be fit satisfactorily with the ‘*two sets of sites*’ binding model. These two models are briefly described below.

#### One set of sites binding model

For a system of one set of identical binding sites, the total heat evolved (or absorbed) during the binding process at the end of the *i*th injection, Q(*i*), is given by Equation (1) [41], [42]:
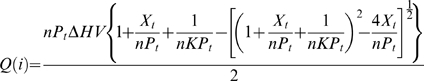
(1)where *n* is the number of binding sites, *P*_t_ is the total protein concentration, *X*_t_ is the total ligand concentration, *V* is the cell volume, *K* is the binding constant and Δ*H* is the binding enthalpy. The heat corresponding to the *i*th injection only, ΔQ(*i*), is equal to the difference between Q(*i*) and Q(*i−1*) and is given by Equation (2), which involves the necessary correction factor for the displaced volume (the injection volume d*Vi*):
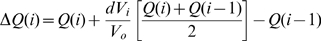
(2)


The ITC unit measures ΔQ(*i*) value for every injection. These values are then fitted to Equations (1) and (2) by a nonlinear least squares method. The fit process involves initial guess of *n*, *K* and Δ*H* which allows calculation of ΔQ(*i*) values as mentioned above for all injections and comparing them with the corresponding experimentally determined values. Based on this comparison the initial guess of *n*, *K* and Δ*H* is improved and the process is repeated till no further significant improvement in the fit can be obtained.

#### Two sets of sites binding model

For a system with two sets of independent binding sites, the total heat evolved (or absorbed) during the binding process at the end of the *i*th injection, Q(*i*), is given by Equation (3) [42]:
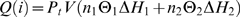
(3)where *n*_1_ and *n*_2_ are the number of binding sites of type 1 and type 2, and Δ*H*_1_ and Δ*H*_2_ are the corresponding enthalpies of binding. Θ_1_ and Θ_2_ are the fractions of the type 1 and type 2 sites that are occupied and are related to the association constants *K*_1_ and *K*_2_, according to equation (4):
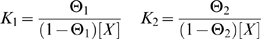
(4)


The heat corresponding to the *i*th injection only, ΔQ(*i*), is equal to the difference between Q(*i*) and Q(*i−1*) and is given by Equation (5), which involves the necessary correction factor for the displaced volume (the injection volume d*V_i_*), according to Equation (2).

The ITC unit measures ΔQ(*i*) value for every injection. These values are then fitted to Equations (2) and (3) by a nonlinear least squares method. The fit process involves initial guess of *n_1_*, *n_2_*, *K_1_*, *K_2_*, Δ*H_1_* and Δ*H_2_* which allows calculation of ΔQ(*i*) values as mentioned above for all injections and comparing them with the corresponding experimentally determined values. Based on this comparison the initial guess of the values of the above parameters is improved and the process is repeated till no further significant improvement in the fit can be obtained.

From the values of *K* and Δ*H*, the thermodynamic parameters, Δ*G* and Δ*S* are calculated according to the basic thermodynamic Equations (5) and (6):
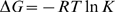
(5)

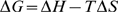
(6)


## Results

### Binding and solubilization of phospholipid membranes by PDC-109

**Figure 1:**
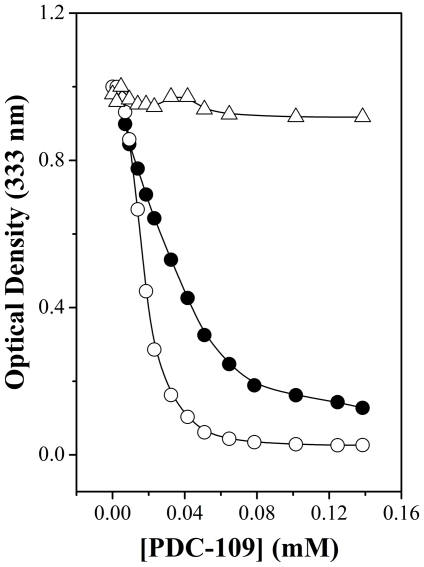
PDC-109 induced solubilization of phospholipid multilamellar vesicles. Solubilization was monitored by turbidimetry by measuring optical density at 333 nm. The phospholipids used are: DMPC (○), DMPG (•) and DMPE (Δ). See text for details.

The binding of PDC-109 to multilamellar vesicles of different phospholipids, namely DMPC, DMPG or DMPE was investigated by turbidimetry monitoring the optical density of the samples at 333 nm as a function of protein concentration as shown in Fig. 1. It is clear from this figure that the turbidity of DMPC vesicles decreases abruptly at about 0.03 mM protein concentration and then levels off (curve 1). In the case of DMPG maximum decrease in turbidity was observed at about 0.06 mM protein concentration (curve 2), whereas turbidity of DMPE vesicles was essentially unaffected even at the highest concentration of PDC-109 employed (curve 3).

### Thermodynamics of PDC-109 binding to phosphatidylcholine unilamellar vesicles

**Figure 2:**
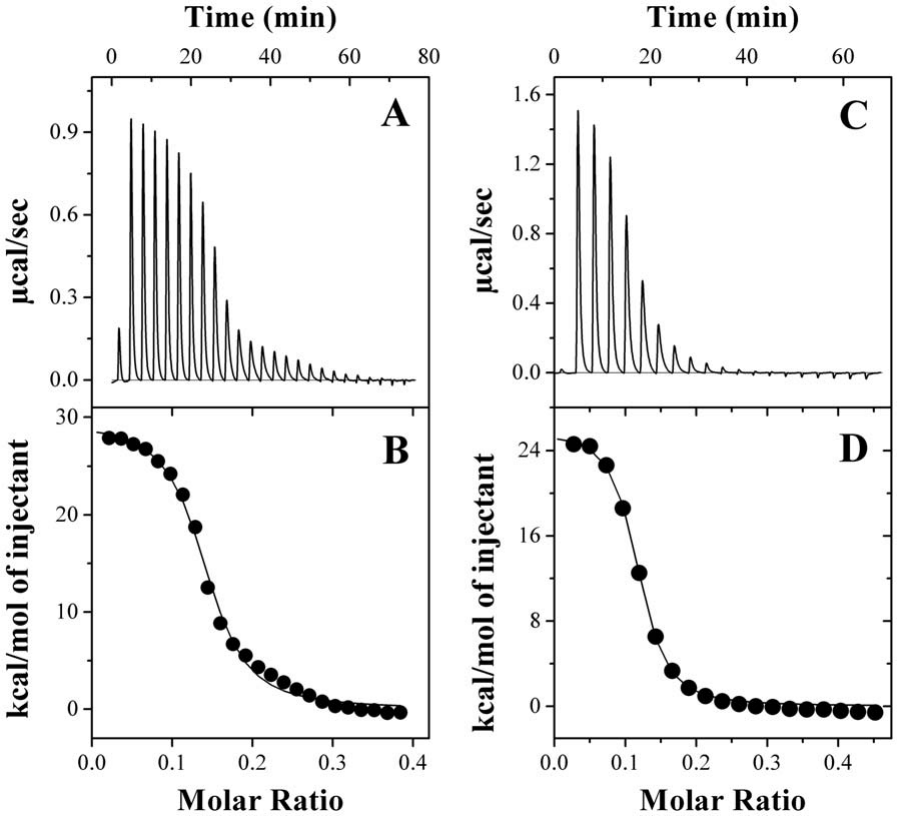
Calorimetric titrations for the binding of PDC-109 to DMPC membranes in the gel phase in the absence and presence of cholesterol. ITC profiles corresponding to the binding of PDC-109 to unilamellar vesicles of DMPC (**A**, **B**) and DMPC/cholesterol (3∶1; mol/mol) (**C**, **D**) at 20°C are shown. Upper panels (**A** & **C**) show the raw data for the titration of phospholipid vesicles with protein and lower panels (**B** & **D**) show the integrated heats of binding obtained from the raw data, after subtracting the heat of dilution. The solid lines in the bottom panels represent the best curve fits to the experimental data, using the *one set of sites* model from MicroCal Origin. See text for further details.

**Figure 3:**
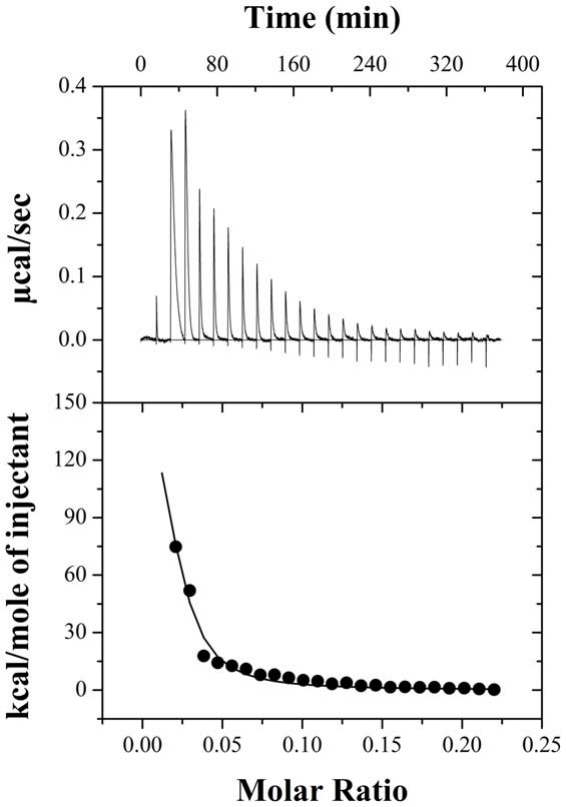
Titration calorimetry for PDC-109 binding to DMPG vesicles. Injection of PDC-109 (250 µM) to the phospholipid vesicles (∼90 µM) at 20°C, yielded an endothermic binding isotherm (**A**). Lower panel (**B**) shows the integrated data obtained from the raw data, after subtracting the heat of dilution. Experimental data were fitted by using the *one set of sites* model from MicroCal Origin.

Thermodynamic parameters characterizing the interaction of PDC-109 with different phospholipids were determined by ITC measurements at various temperatures below the gel-liquid crystalline phase transition. To minimize the effect of temperature variation that occurs during the injection on their phase structure, the phospholipids were taken in the calorimeter cell and PDC-109 was added through the syringe. Figs. 2A and 2B show typical ITC profiles for the binding of the protein to DMPC unilamellar vesicles at 20°C. The titration profile in Fig. 2A shows that injection of 5 µL aliquiots of PDC-109 into DMPC suspension gives large endothermic heats of binding, which decrease in magnitude with subsequent injections, showing saturation behavior. The data could be best fitted by a nonlinear least squares approach to the ‘*one set of sites*’ binding model, which yielded the association constant (*K*_a_), stoichiometry of binding (*n*), and the thermodynamic parameters, enthalpy of binding (Δ*H*), entropy of binding (Δ*S*) and free energy of binding (Δ*G*). Four independent measurements at 20°C yielded the values of *K*_a_, *n*, Δ*H*, Δ*S*, and Δ*G* as 4.1 (±1.5)×10^5^ M^−1^, 7.2 (±0.3), 4.5 (±0.5) kcal.mol^−1^, 40.8 (±1.8) cal.mol^−1^.K^−1^, and −7.52 kcal.mol^−1^, respectively. These values as well as the values obtained at other temperatures for the PDC-109/DMPC interaction are listed in Table 1. Similar titrations were carried out with DMPG and DPPC, and representative ITC profiles corresponding to the titration of these two lipids with PDC-109 are presented in Fig. 3 and Fig. S2, respectively. Values of *K*_a_, *n*, Δ*G*, Δ*H* and Δ*S* derived from the analysis of the titration data corresponding to the binding of PDC-109 to these lipids are also listed in Table 1. These values as well as those given in all other tables correspond to the averages obtained from 2–4 independent titrations and standard deviations are indicated in parentheses. The observed binding constant for the interaction of PDC-109 with DMPG is smaller than the *K*_a_ values obtained for the binding of the choline phospholipids (see Table 1), reflecting its lower affinity for PDC-109. Thermodynamic parameters obtained for the interaction of DMPC, DPPC and DMPG to PDC-109 indicate that the binding is stabilized by entropic factors, with negative contribution from enthalpy of binding. A few titrations were also carried out with DMPC or DPPC in the liquid crystalline phase; however, these preliminary experiments yielded complex isotherms and a representative isotherm corresponding to the binding of PDC-109 to DMPC vesicles at 30°C is given in Fig. S3. These isotherms could not be analyzed satisfactorily with the binding models available in the Origin ITC analysis software. Hence no further experiments were carried out in the fluid phase with these lipids.

The data presented in Table 1 show that the number of lipid molecules corresponding to each molecule of PDC-109 decreases with increasing temperature whereas the binding enthalpy and entropy increase with temperature. However, the enthalpy values did not exhibit clear linear temperature dependence; hence, it was not possible to obtain the heat capacity changes associated with the PDC-109/phospholipid interaction.

### Effect of cholesterol on the binding of PDC-109 to DMPC membranes

Binding isotherms for the interaction of PDC-109 with DMPC/cholesterol (3∶1; mol/mol) mixtures were also characterized by endothermic heats of binding which decreased in magnitude with successive injections until saturation was achieved (Fig. 2C). The data could be best analyzed by a nonlinear least squares fit to the ‘*one set of sites*’ binding model in the Origin ITC data analysis software. As summarized in Table 2, average values of *K*_a_, *n*, Δ*G*, Δ*H* and Δ*S* obtained for the interaction of PDC-109 with DMPC membranes containing 25 mol% cholesterol at 20°C are 5.8 (±1.9)×10^5^ M^−1^, 8.7 (±0.3), −7.73 kcal.mol^−1^, 2.6 (±0.3) kcal.mol^−1^, and 35.1 (±0.3) cal.mol^−1^.K^−1^, respectively. The binding enthalpy for this interaction increases initially with increase in temperature, but decreases with further increase in temperature (Table 2). The ITC profiles were found to be endothermic between 10 and 30°C for the binding of PDC-109 to DMPC/cholesterol mixtures, in contrast to membranes containing DMPC alone, which yielded endothermic profiles in the gel phase and complex binding isotherms (containing both endothermic and exothermic components) in the liquid crystalline phase.

**Table 2:** Thermodynamic parameters obtained by isothermal titration calorimetry for the binding of PDC-109 to DMPC/cholesterol (3∶1; mol/mol) mixture.

Temperature (°C)	*n*	*K*_a_×10^−5^ (M^−1^)	Δ*G*(kcal.mol^−1^)	Δ*H* (kcal.mol^−1^)	Δ*S* (cal.mol^−1^.K^−1^)
10*	21.5 (±0.9)	1.4 (±0.4)	−6.66	1.2 (±0.1)	27.6
15	10.9 (±0.2)	2.8 (±0.2)	−7.18	2.3 (±0.1)	32.8 (±0.3)
20	8.7±(0.3)	5.8 (±1.9)	−7.73	2.6 (±0.3)	35.1 (±0.3)
25	7.4±(0.1)	4.5 (±0.5)	−7.71	2.5 (±0.1)	34.4 (±0.2)
30	6.2±(0.1)	4.1 (±0.2)	−7.78	1.7 (±0.1)	31.1 (±0.1)

*Data from a single measurement.

Values reported are averages of results obtained from 2–3 independent experiments with standard deviations indicated in parentheses.

### Thermodynamics of PDC-109 binding to lysophosphotidylcholine micelles

**Figure 4:**
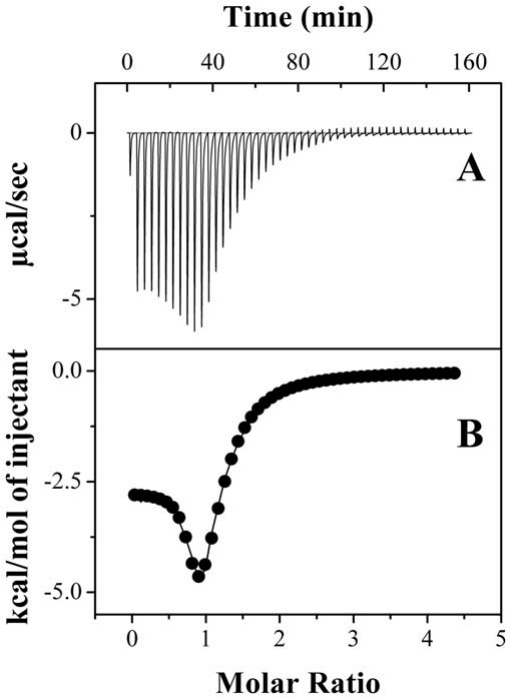
Binding of PDC-109 with Lyso-PC micelles. **A**) Raw data for the titration of 450 µM protein with 10.88 mM Lyso-PC at 25°C. **B**) Integrated heats of binding obtained from the raw data, after subtracting the heat of dilution. The solid line in **B** represents the best curve fit to the experimental data, using the *two sets of sites* model from MicroCal Origin.

An isotherm for the binding of Lyso-PC to PDC-109 at 25°C is shown in Fig. 4. The raw titration data shown in the upper panel (A) could be best fitted to the ‘*two sets of sites*’ binding model in the Origin software and the endothermic heats of binding corresponding to the successive injections together with the fit obtained (shown as a solid line) are given in the lower panel (B). This analysis suggests two kinds of binding interactions between the protein and Lyso-PC, one characterized by a higher affinity and the other with a lower affinity. Two independent titrations yielded the following average parameters (with standard deviations indicated in the parentheses) for the high affinity interaction: stoichiometry (the number of Lyso-PC molecules bound to each molecule of PDC-109, *n*) = 0.9 (±0.1), association constant (*K*_a_) = 7.0 (±0.2)×10^5^ M^−1^, enthalpy of binding (Δ*H*) = −2.6 (±0.1) kcal.mol^−1^ and entropy of binding (Δ*S*) = 17.9 (±0.4) cal.mol^−1^.K^−1^. The corresponding values obtained for the low-affinity interaction are: *n* = 3.5, *K*_a_ = 1.2 (±0.1)×10^4^ M^−1^, Δ*H* = −13.7 (±0.6) kcal.mol^−1^, and Δ*S* = −2.6 (±0.1) cal.mol^−1^.K^−1^. It is interesting to note that while enthalpy of binding is favorable for both types of interactions, entropic factors are favorable only for the high-affinity interaction (Δ*S* = +ve), with the low-affinity interaction being characterized by a large unfavorable contribution from the binding entropy (Δ*S* = −ve).

### Effect of pH on the interaction of PDC-109 with DMPC

The effect of pH on the interaction of PDC-109 with DMPC vesicles was studied at a fixed temperature by ITC experiments. Representative ITC profiles and the corresponding fits for measurements performed at pH 7.4 and pH 9.0 are shown in Fig. S4 and the thermodynamic parameters obtained are listed in Table 3. Measurements could be performed only in the narrow pH range of 7.4 and 9.0 since the protein tended to precipitate at low pH and ITC profiles above pH 9.0 were not consistent. The results obtained indicate that the association constant for DMPC increases approximately two-fold at higher pH (Table 3).

**Table 3:** Effect of pH on thermodynamic parameters obtained by isothermal titration calorimetry for PDC-109 binding to DMPC unilamellar vesicles.

pH	*n*	*K*_a_×10^−5^ (M^−1^)	Δ*G* (kcal.mol^−1^)	Δ*H* (kcal.mol^−1^)	Δ*S* (cal.mol^−1^.K^−1^)
7.4	12.3 (±1.3)	1.7 (±0.5)	−6.89	3.1 (±0.2)	34.6 (±0.14)
8.0	18.0 (±6.9)	4.5 (±1.6)	−7.45	2.7 (±0.2)	33.0 (±2.4)
9.0	6.7 (±1.9)	5.1 (±0.7)	−7.52	3.2 (±0.5)	37.3 (±2.0)

Values reported are averages of results obtained from 2–3 independent experiments with standard deviations indicated in parentheses. Temperature = 15°C.

## Discussion

In order to characterize the energetics that define the interaction of PDC-109 with phospholipid membranes, ITC experiments have been carried out in the present study. This method has been widely used in recent times for investigating the interaction of peptides and proteins with membranes [43]–[46]. Previously, the interaction of PDC-109 with POPC membranes was studied by ITC and FTIR and the effect of POPG, POPE and cholesterol on this interaction were investigated [38]. It was observed that lipid composition has a considerable influence on the binding, which was observed to be endothermic at low temperature and exothermic at high temperature. However, in the above study the calorimetric titrations were not continued till saturation binding and the data obtained were analysed by Scatchard plots, which yielded only the stoichiometry and binding constants. In the present study, we have investigated the binding of PDC-109 to vesicles of DMPC, DPPC, DMPG and DMPC/cholesterol by calorimetric titrations performed at different temperatures. In addition, the effect of pH on the interaction of this protein with DMPC was investigated. Finally, the interaction of PDC-109 with Lyso-PC micelles was also investigated by ITC. In each case, the titrations were performed till saturation binding was achieved so that the data could be analysed more thoroughly, which yielded the stoichiometry (*n*) and the various thermodynamic parameters associated with the binding, viz., *K*_a_, Δ*H*, Δ*S* and Δ*G*.

The enthalpies and entropies for the binding of PDC-109 to DMPC, DPPC and DMPG membranes are all positive (Table 1), clearly indicating that the binding is entropically favored. Such entropic control of the binding process indicates that hydrophobic interactions play a predominant role in the association between PDC-109 and phospholipid membranes. This is in agreement with previous studies employing spin-label ESR spectroscopy, hydropathy analysis and fluorescence spectroscopy, which indicated that short segments of the protein containing predominantly hydrophobic amino acid residues may be embedded in the hydrophobic interior of DMPC membranes [23], [27], [47]. Entropically driven endothermic binding of peptides and proteins to lipid membranes has been reported in a number of studies. For example, binding of the proline utilization A flavoenzyme from *Bradyrhizobium japonicum* to *E. coli* polar vesicles is associated with a positive enthalpy of binding at 25°C, whereas the binding at 5°C was observed to be exothermic [48]. Binding of the β-amyloid peptide, Aβ (1–40) to zwitterionic lipids at 25°C was reported to be associated with positive enthalpy of binding [49]. Binding of a rationally designed analogue of gramicidin S (GS14*d*K4) to negatively charged lipid membranes at 25°C has been shown to be endothermic with a positive change in entropy [46].

It is pertinent to discuss the involvement of specific amino acid residues of PDC-109 in its interaction with membranes made up of different lipids. Although ITC does not provide any information on this aspect, some information is available in this regard from previous studies. Single-crystal X-ray diffraction studies by Calvete and coworkers [22] revealed that each FnII domain of PDC-109 binds the phosphorylcholine head group of choline phospholipids and this interaction involves Tyr-30, Trp-47 and Tyr-54 (first Fn-II domain) and Tyr-75, Tyr-100 and Trp-93 (second Fn-II domain). In some of the molecules, Trp-93 and Tyr-108 may also take part in choline phospholipid binding [22]. Fluorescence spectroscopic studies coupled with hydropathy analysis suggested that when PDC-109 interacts with choline-containing phospholipids such as DMPC and DPPC segments of the protein containing Trp-90 is likely to be embedded in the membrane interior [23], [27]. Also, some of the cholesterol recognition/interaction amino acid consensus (CRAC) sequences at positions 50–57 (LDADYVGR), 55–64 (VGRWKYCAQR), 70–78 (VFPFIYGGK) and 97–103 (LSPNYDK) in PDC-109 [50] may directly interact with cholesterol in membranes. It has been proposed that the N-terminal Leu/Val residue in these sequences interacts with the hydrophobic side chain of cholesterol whereas the tyrosine residue in the middle forms a hydrogen bond with the hydroxyl moiety of cholesterol [50], [51].

**Figure 5:**
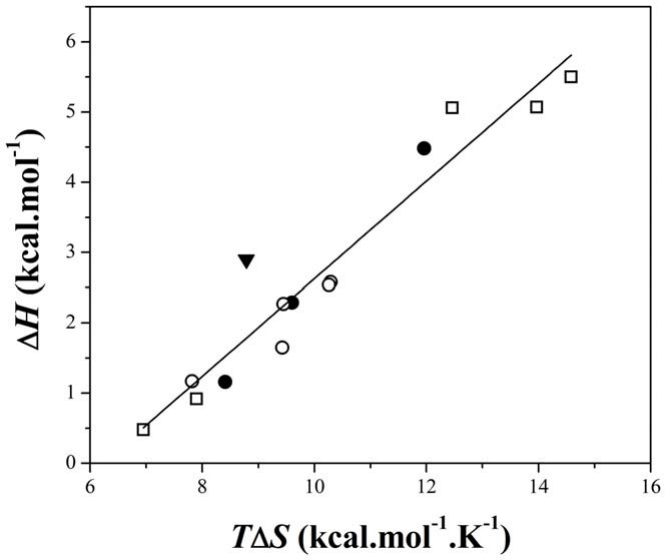
Enthalpy-entropy compensation plot. Data are shown for the interaction of PDC-109 with phospholipid vesicles made up of DMPC (•), DPPC (□), DMPC/cholesterol (O) and DMPG (▾). The straight line corresponds to a linear least squares fit (slope = 0.69).

An enthalpy-entropy compensation plot (Δ*H* versus *T*Δ*S*) for the binding of PDC-109 to membranes made up of DMPC, DPPC, DMPC/cholesterol (3∶1; mol/mol) as well as DMPG at different temperatures is shown in Fig. 5. It is clear from the linearity of the data presented in this figure that binding of PDC-109 to phospholipid or phospholipid/cholesterol membranes is associated with a close compensation of enthalpy and entropy. An exact compensation of enthalpy by entropy would yield a slope of 1.0 [52], [53], and deviation of this would indicate the predominance of one of these factors over the other. For slope >1.0, binding is governed primarily by enthalpy of binding, whereas a slope <1.0 indicates a process that is predominantly entropy-driven [52], [54]. The observed slope of 0.69 for the fit in Fig. 5 strongly suggests that binding of PDC-109 to lipid membranes is driven predominantly by entropic forces. This is completely in agreement with the thermodynamic data obtained in the present study as well as the results obtained from surface plasmon resonance studies on the PDC-109/phospholipid interaction, which showed that the binding is governed by favorable entropic forces with negative contribution from enthalpy of binding [25]. However, the binding strengths (association constants) for the interaction of different phospholipids with PDC-109 obtained in this study by ITC are at variance with the results of our earlier surface plasmon resonance (SPR) studies [25], although the relative affinities for different phospholipids are largely consistent between the two studies. This difference may arise due to the differences in the structure of membranes used in these two studies. In the SPR studies, hybrid bilayers containing a phospholipid monolayer deposited on an alkanethiol coated sensor chip (wherein the lipid layer is planar) were employed, whereas in the present study small unilamellar vesicles, which are intrinsically highly curved, have been used.

Although complex isotherms were obtained for the binding of PDC-109 to DMPC vesicles in the fluid phase, upon cholesterol incorporation simple endothermic binding isotherms were obtained even at temperatures corresponding to the liquid crystalline phase of DMPC. These results are consistent with the abolition of the gel-fluid phase transition and the rigidification of liquid crystalline phase of DMPC membranes by cholesterol [55], since endothermic binding isotherms were obtained with DMPC alone only in the gel phase region where the lipid acyl chains are rigid and tightly packed.

For phosphatidylcholines in the absence and in the presence of cholesterol, the number of binding sites is found to decrease with temperature, i.e., the number of lipid molecules associated with each molecule of PDC-109 decreases as the temperature is increased (Tables 1 and 2). It is well known that the cross-sectional area of lipids increases with increase in temperature [56]. Assuming that the area of the protein which interacts with the lipid membrane does not change significantly in the temperature range studied, it is expected that the number of lipid molecules that would be associated with the protein would decrease as the temperature is increased. The estimated stoichiometry may also be affected by temperature-induced alterations in the aggregation state of PDC-109, which is known to exist as a polydisperse aggregate in solution [57].

In the presence of excess water, Lyso-PC forms micelles that coexist with dissolved Lyso-PC molecules. The two types of interactions observed for the binding of PDC-109 to Lyso-PC can therefore be interpreted as due to the binding of the protein with the micelles and free Lyso-PC, respectively. Although the high-affinity interaction is aided by both entropic and enthalpic contributions, the entropic contribution is more dominant. On the other hand the low-affinity interaction is enthalpically driven, with negative contribution from entropy of binding. Since the interaction of PDC-109 with different phospholipid vesicles are all governed by positive entropic contribution at all temperatures, it is most likely that the high affinity interaction, which also has a significantly large positive entropy of binding, corresponds to the binding of PDC-109 to Lyso-PC micelles. The weaker interaction will then correspond to the association of free Lyso-PC molecules with PDC-109.

Changing the pH of the medium is known to induce conformational changes in the secondary and tertiary structures of proteins [58]. Since PDC-109 is present in a slightly basic environment in the seminal plasma, it is of interest to study the effect of pH on its interaction with membranes containing the major plasma membrane lipid phosphatidylcholine, for which this protein exhibits the highest affinity. The thermodynamic data presented in Table 3 shows that the association constant for the binding of PDC-109 to DMPC vesicles increases approximately 2-fold when the pH was increased from 7.4 to 9.0, which could be physiologically relevant in view of the basic nature of the seminal plasma of mammals.

In summary, the energetics of interaction of PDC-109 with phospholipids has been investigated by isothermal titration calorimetry. It has been found that the binding of PDC-109 to gel phase lipids is endothermic in nature and is governed by a positive entropic contribution. The binding strength of PDC-109 for choline containing phospholipids was observed to be higher than that of other phospholipids such as DMPG and DMPE, which is consistent with the higher specificity of PDC-109 for the choline phospholipids. The affinity of PDC-109 for phosphatidylcholine was found to be higher at higher pH, which is physiologically relevant in view of the basic nature of the seminal plasma. Overall, the present study leads to a better understanding of the interaction of PDC-109 with phospholipid membranes at the molecular level, which is relevant to understanding the interaction of PDC-109 with sperm plasma membrane during sperm capacitation, *in vivo*.

### Supporting Information Available

Additional information relevant to this article, containing Figures S1, S2, S3, S4 is given as Supporting Information.

## Supporting Information

Figure S1 **SDS-PAGE of PDC-109.** Lane 1, molecular weight markers; lane 2, PDC-109. The Mr values of the standard proteins (in kDa) are indicated on the left.
(TIF)

Figure S2 **Calorimetric titration for the binding of PDC-109 to DPPC unilamellar vesicles in the gel phase at 36°C.** Upper panel shows the raw data for the titration of phospholipid vesicles with protein and the lower panel shows the integrated heats of binding obtained from the raw data, after subtracting the heats of dilution. The solid line in the lower panel represents the best curve fit to the experimental data, using the *one set of sites* model from MicroCal Origin.
(TIF)

Figure S3 **Calorimetric titration for the binding of PDC-109 to DMPC unilamellar vesicles in the liquid crystalline phase at 30°C.** Upper panel shows the raw data for the titration of phospholipid vesicles with protein and the lower panel shows the integrated heats of binding obtained from the raw data, after subtracting the heat of dilution.
(TIF)

Figure S4 **Effect of pH on the binding of PDC-109 to DMPC membranes.** Representative ITC profiles are given for titrations performed at pH 7.4 (left panel) and 9.0 (right panel). The parameters obtained from the fits are indicated in the boxes given in the figure. The data given in Table 3 in the main manuscript correspond to average values from 2–3 independent titrations.
(TIF)
