# Supplementary figures and images for: Isothermal Titration Calorimetric Studies on the Interaction of the Major Bovine Seminal Plasma Protein, PDC-109 with Phospholipid Membranes
Source: PLoS One. 2011 Oct 14;6(10):e25993. doi: 10.1371/journal.pone.0025993 (PMC3193528; doi:10.1371/journal.pone.0025993)

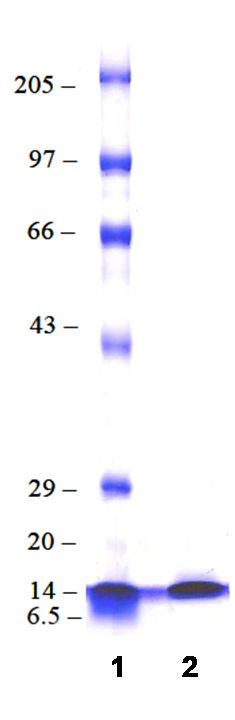

Supplement: Figure S1 — SDS-PAGE of PDC-109. Lane 1, molecular weight markers; lane 2, PDC-109. The Mr values of the standard proteins (in kDa) are indicated on the left. (TIF) [file pone.0025993.s001.tif]

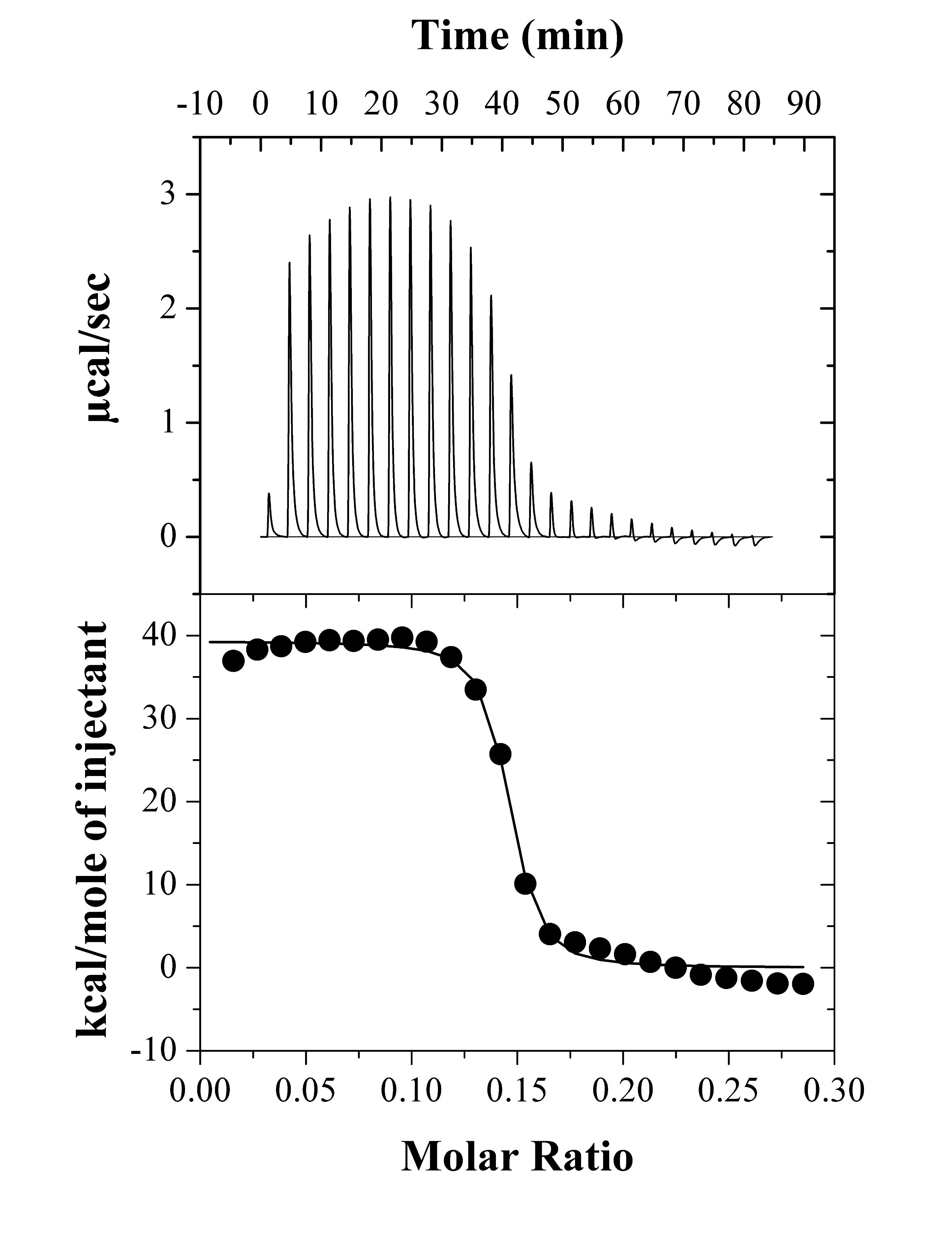

Supplement: Figure S2 — Calorimetric titration for the binding of PDC-109 to DPPC unilamellar vesicles in the gel phase at 36°C. Upper panel shows the raw data for the titration of phospholipid vesicles with protein and the lower panel shows the integrated heats of binding obtained from the raw data, after subtracting the heats of dilution. The solid line in the lower panel represents the best curve fit to the experimental data, using the one set of sites model from MicroCal Origin. (TIF) [file pone.0025993.s002.tif]

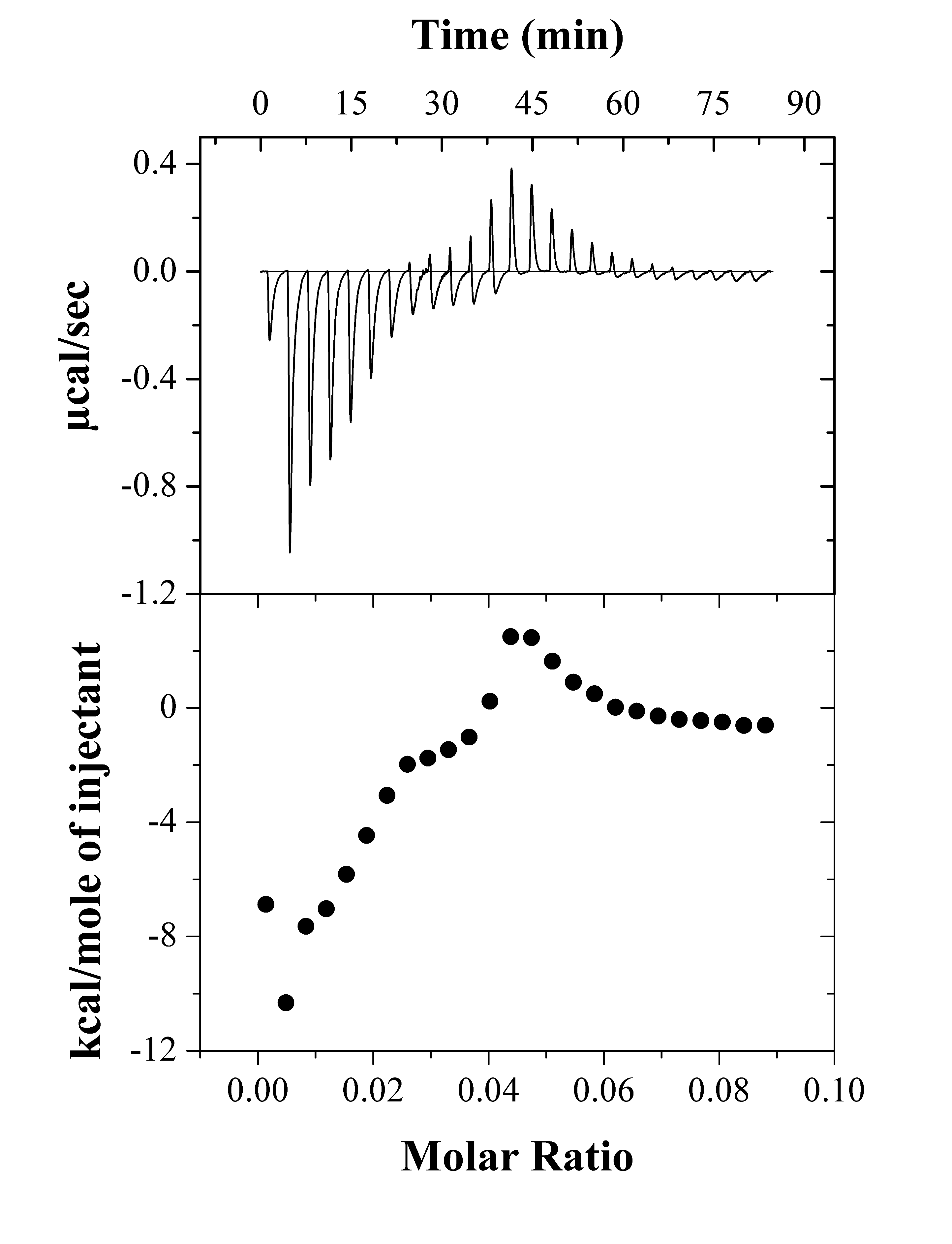

Supplement: Figure S3 — Calorimetric titration for the binding of PDC-109 to DMPC unilamellar vesicles in the liquid crystalline phase at 30°C. Upper panel shows the raw data for the titration of phospholipid vesicles with protein and the lower panel shows the integrated heats of binding obtained from the raw data, after subtracting the heat of dilution. (TIF) [file pone.0025993.s003.tif]

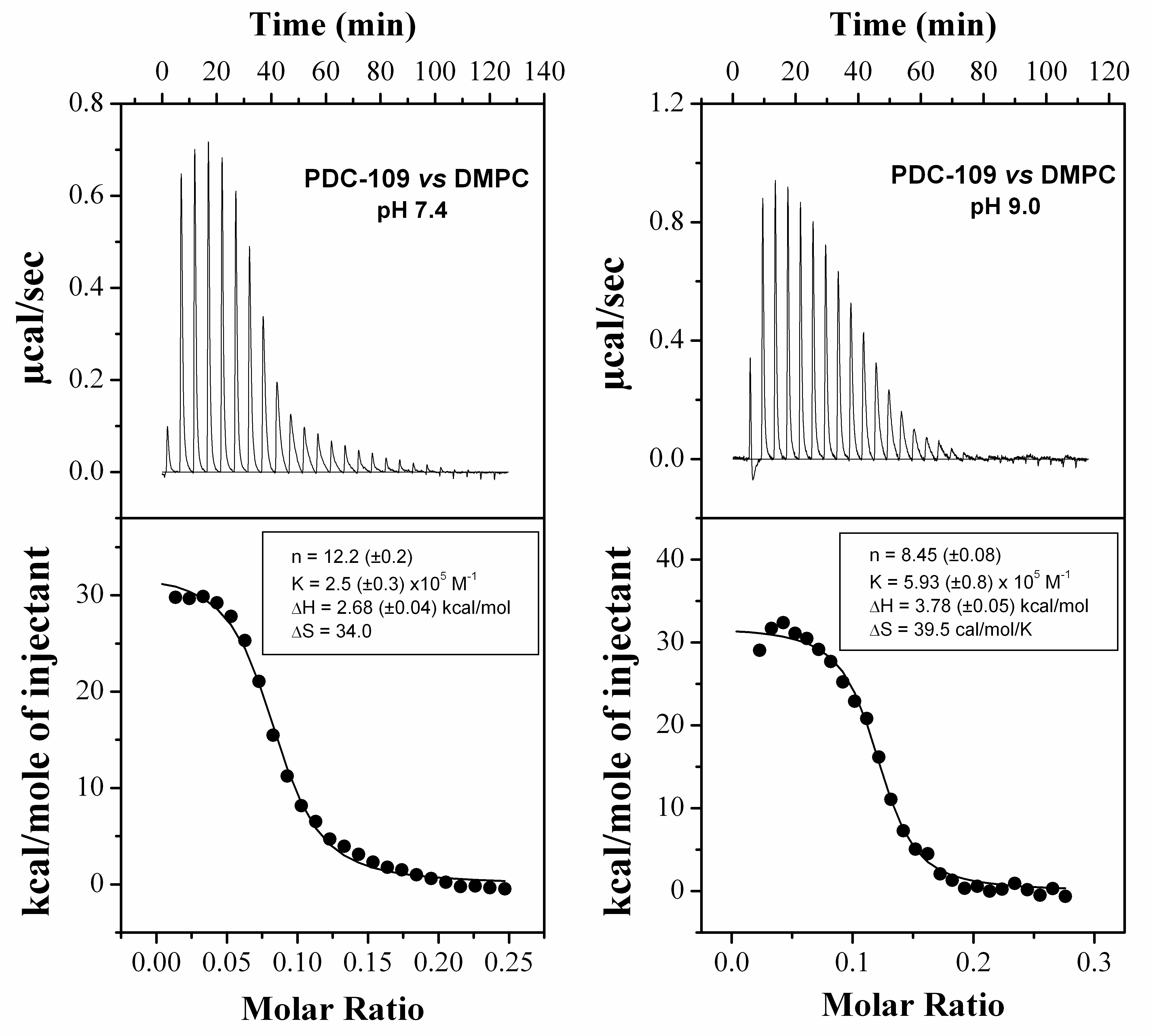

Supplement: Figure S4 — Effect of pH on the binding of PDC-109 to DMPC membranes. Representative ITC profiles are given for titrations performed at pH 7.4 (left panel) and 9.0 (right panel). The parameters obtained from the fits are indicated in the boxes given in the figure. The data given in Table 3 in the main manuscript correspond to average values from 2–3 independent titrations. (TIF) [file pone.0025993.s004.tif]
